# The Use of IMMUs in a Water Environment: Instrument Validation and Application of 3D Multi-Body Kinematic Analysis in Medicine and Sport

**DOI:** 10.3390/s17040927

**Published:** 2017-04-22

**Authors:** Anna Lisa Mangia, Matteo Cortesi, Silvia Fantozzi, Andrea Giovanardi, Davide Borra, Giorgio Gatta

**Affiliations:** 1 Health Sciences and Technologies—Interdepartmental Centre for Industrial Research, University of Bologna, Via Tolara di Sopra, 50, 40064 Ozzano dell’Emilia, Italy; annalisa.mangia2@unibo.it; 2 Department for Life Quality Studies, University of Bologna, C.so D’Augusto, 237, 47921 Rimini, Italy; m.cortesi@unibo.it (M.C.); giorgio.gatta@unibo.it (G.G.); 3 Department of Electrical, Electronic and Information Engineering, University of Bologna, Viale del Risorgimento, 2, 40136 Bologna, Italy; davide.borra@studio.unibo.it; 4 School of Pharmacy, Biotechnology and Sport Science, University of Bologna, Viale Berti Pichat 10, 40127 Bologna, Italy; a.giova83@gmail.com

**Keywords:** IMMU, gait, swimming, water, biomechanics, performance analysis, sports science, stroke analysis

## Abstract

The aims of the present study were the instrumental validation of inertial-magnetic measurements units (IMMUs) in water, and the description of their use in clinical and sports aquatic applications applying customized 3D multi-body models. Firstly, several tests were performed to map the magnetic field in the swimming pool and to identify the best volume for experimental test acquisition with a mean dynamic orientation error lower than 5°. Successively, the gait and the swimming analyses were explored in terms of spatiotemporal and joint kinematics variables. The extraction of only spatiotemporal parameters highlighted several critical issues and the joint kinematic information has shown to be an added value for both rehabilitative and sport training purposes. Furthermore, 3D joint kinematics applied using the IMMUs provided similar quantitative information than that of more expensive and bulky systems but with a simpler and faster setup preparation, a lower time consuming processing phase, as well as the possibility to record and analyze a higher number of strides/strokes without limitations imposed by the cameras.

## 1. Introduction

Water has always been believed to promote the quality of life and has therefore been used in the clinical and sports fields. The analysis of movements in water helps in increasing the efficacy of therapeutic treatments, the efficiency of techniques in aquatic sports [1], and in characterizing the technical errors or performance compliance with the training process [2].

In clinical applications, the traditional methods applied to investigate the biomechanical parameters of underwater (UW) locomotion are semi-subjective, i.e., an expert examines visually, through special windows, the quality of the movement performed by the patient [3]. Similarly, in aquatic sports, the coaches observe the swimmer’s movement mainly using qualitative methods based on naked eye observation, stopwatches, and their own experience and knowledge [4]. A key advantage is that this approach is both low cost and easy to implement on a large number of patients and athletes. On the other hand, a quantitative analysis can improve the understanding of patients/swimmers’ biomechanics, enabling the improvement of their therapy/performance [1,5,6].

Traditionally, methods for the collection of quantitative information about movement in UW settings are video-based [7,8,9,10,11]. Cameras positioned above and/or below the level of water acquire images to obtain speed, position, and other biomechanical variables of the anatomical landmarks of interest [12]. Video-based analysis is a reliable and accurate method for UW motion analysis in rehabilitative activities and sports. However, this type of analysis involves several drawbacks, like (i) increased time-consuming installation, calibration, and data processing due to the extensive number of synchronized cameras needed for a 3D kinematic analysis [13]; (ii) limited field of view that allows for the analysis of few cycles of stride/stroke resulting in a restriction of the exercise duration evaluation [14]; (iii) turbulences, refraction of light, and parallax error at the water-air interface, decreasing accuracy [15]; (iv) off-line analysis involving a delay in the feedbacks provided to the patient/athlete with a negative effect on motor learning [16]. For these reasons, the biomechanics of human movements in water have not been adequately explored [17].

Technology advances in the design of miniaturized, low-cost, and low-power sensors have recently enabled their use for a wide variety of biomedical applications. In particular, Micro Electro-Mechanical Systems (MEMS) accelerometers, gyroscopes, and magnetometers, are strategic for human movement analysis, because of their characteristics of portability and the useful kinematic information they provide. Indeed, a sensor fusion approach allows the computation of an optimized orientation estimation [18]. The potential of such technology for movement analysis has become increasingly widespread [19,20,21,22]. However, such an approach still needs to be fully exploited for a correct and accurate use in UW movement analysis.

In the last fifteen years, many research groups have worked with inertial-magnetic measurements units (IMMUs) in aquatic environments. Considering the clinical context, despite the extensive work of validation for biomechanical analysis of walking on land using IMMUs [23,24,25,26], only a few studies tackled a similar analysis in water to measure kinetic and kinematic variables. Among kinetic variables, peak velocities and the power of UW motion were obtained using a single unit [27,28]. More recently, Marinho-Buzelli et al. [29] examined the postural control in water through trunk acceleration parameters using two body-worn inertial sensors. With regards to sport contexts, most studies have focused on the temporal phase detection of front crawl swimming. Among them, Oghi [30] was the first who analyzed the stroke parameters using a single integrated unit placed on the wrist. Successively, several studies have analyzed the temporal stroke parameters and the speed profile using single or multiple sensors, in one case also integrated with video [31]. However, the extraction of only spatiotemporal parameters is not sufficient to highlight differences between patients/athletes’ biomechanics, and a 3D multi-body kinematics analysis is required to better quantify their motor patterns and to improve the outcome of their therapy/performance [32,33].

The extensive use in water was motivated by some advantages with respect to the video-based method. The biomechanical analysis using inertial sensors, different from the video-based method, allows for a continuous data collection, a simple and fast set-up, and has the advantage of providing results in real-time during the rehabilitative/training session. Furthermore, similar to video cameras, the use of this technology does not impact the ability of the patient/athlete to naturally perform the movement. However, inherent motion analysis errors using IMMUs are still unresolved as (i) the fixed bias refers to the offset of the accelerometer signal from the true value; (ii) scale factor errors (iii) cross-coupling erroneous output due to the accelerometer sensibility and (iv) the gyroscope drift [34], influencing the accuracy of the orientation estimation.

The accuracy of the orientation estimation with an IMMU have been previously investigated out of water using static and dynamic validations, mostly under manually generated conditions [35,36,37,38] and rarely under controlled conditions [39,40,41,42]. These studies pointed out critical aspects in measuring relative orientation with IMMUs, reporting errors up to 11.7°. One of the main assumptions in the sensors’ fusion algorithms for IMMU orientation estimation is the homogeneity of the earth magnetic field. Typically, the laboratories used for validation of this new equipment present field irregularities caused by construction iron in the floors, walls, and ceilings, or other technical equipment [43,44]. Likewise, in a swimming pool, the pool’s side and the floor could induce inhomogeneities, and the distortion of the magnetic field in the water should be characterized. Therefore, it is suggested to map the measurement volume to determine its ferromagnetic characteristics prior to the planned experiments [44]. Also, to explore the potential of systems of measurement based on inertial sensors, and to move towards their systematic use, the definition of specific acquisition protocols is essential. These protocols depend on the body segments under investigation, the particular purpose of the movement assessment, as well as the specific setting in which the tests are performed. Considering this last factor, the previously described validation tests [44] were all performed in a dry land (DL) setting only.

The aim of the present paper is to provide an instrumental validation of IMMUs in water and to describe the use of IMMUs for multi-body joint kinematics in clinical and sport applications.

The remainder of the paper includes: (i) the instrumental validation of IMMUs in the water setting and comparison with the dry setting; and (ii) a broad description regarding the two main applications of biomechanical analysis using IMMUs, walking and swimming. A discussion of the experimental results obtained by the proposed studies is provided to highlight the advantages and limitations of multiple IMMU sensors for multi-body joint kinematic analysis.

## 2. Materials and Methods

### 2.1. Instrument Validation

Several tests to evaluate the accuracy of the sensors in water and the influence of magnetic noise on the magnetometers in the swimming pool were implemented and are hereinafter described. The following tests were performed, in both DL and UW settings: (i) Magnetic field mapping; and (ii) Dynamic orientation accuracy.

#### 2.1.1. Sensors

A commercial system (OPAL, APDM, USA), with 8 IMMUs was used (Table 1). Each IMMU integrates a three axes accelerometer, a three axes gyroscope, and a three axes magnetometer. These IMMUs had small dimensions (48.4 mm long × 36.5 mm high × 13.4 mm wide), low power, and low weight (<22 g), with high internal storage (8 Gb), long battery life, and wireless data transmission. The acquisition frequency of each IMMU was set to 128 Hz and the accelerometer’s full scale was set to ±6 g.

The IMMUs were inserted into round plastic waterproofed boxes during the UW setting tests.

#### 2.1.2. Orientation Algorithm

A Madgwick filter was used for the estimation of orientation [18]. The innovative aspects of this filter include a low computational load, a single adjustable parameter defined by observable system characteristics (named β), an analytically derived and optimized gradient descent algorithm enabling performance at low sampling rates, an on-line magnetic distortion compensation algorithm, and gyroscope bias drift compensation. The accelerometer’s signals and the magnetometer’s signals are combined to generate the settable value gain (β) that determines how much of this combination is desirable to compensate for the gyroscope biases.

#### 2.1.3. Magnetic Field Mapping

This section focuses on the magnetic field calibration, given the need to use the magnetometer’s output in 3D kinematics measures. More specifically, the distortions of the magnetic field effects on the accuracy of the orientation estimation in a standard motion lab (DL setting) and in a swimming pool (UW setting) were investigated.

**Figure 1:**
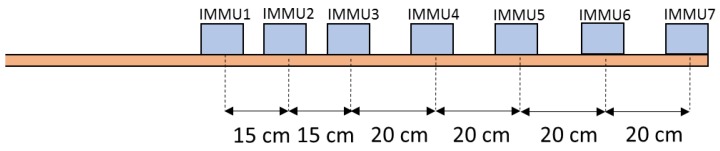
Representation of the wooden bar with the 7 IMMUs (IMMU1–IMMU7) positioned as shown in the figure.

The experimental setup included 7 IMMUs (IMMU1–IMMU7) positioned on a wooden bar, as shown in Figure 1, with the aim of evaluating the influence of a magnetic field distortion introduced by a ferrous object on the accuracy of the orientation estimation as a function of the IMMU-object distance.

Four different tests were performed in static conditions to investigate: (A) the repeatability of the orientation estimation in the pool; (B) the effect of the poolside (UW); (C) the effect of a ferrous object in the laboratory (DL); (D) the effect of a ferrous object in the pool (UW). All the acquisitions performed during the tests lasted 15 s.

In test A, the wooden bar was placed vertically and parallel to the poolside (IMMU7 above the bottom of the pool) at a distance of 25 cm from the poolside and 10 static tests were recorded with the aim of evaluating the orientation estimation repeatability in the pool (UW).

In test B, the wooden bar was placed vertically and parallel to the poolside (IMMU7 above the bottom of the pool) and it was gradually positioned at different decreasing distances from the poolside: 200, 150, 100, 85, 70, 55, 40, 25, 20, 15, 10, 5, and 3 cm. Test B was repeated in three positions along the pool lane: 250, 750, and 1250 cm from the pool side that correspond with the beginning, the midpoint, and the end of the walking test described in the following.

In test C, a metal bar was placed horizontally on the floor and the wooden bar with the IMMUs, placed parallel to the first, was gradually positioned at different decreasing distances from the metal bar: 150, 100, 85, 70, 55, 40, 25, 20, 15, 10, 5, and 3 cm. Because of the smaller size of the metal bar with respect to that of the wooden bar, only 6 units were evaluated (IMMU1–IMMU6). Test D was equal to test C but it was performed in the pool (UW), rather than in DL settings. In test D, the wooden bar was secured with non-ferrous material objects. Both the tests C and D were performed at 30 cm from the floor and from the bottom of the pool, respectively. Tests C and D were repeated three times each.

Given the static nature of the test, we computed the average orientation within the selected time window using a Madgwick filter with β = 0.312, the value optimized for the gait analysis [32]. The orientation of each IMMU was computed and expressed in terms of Euler angles (*alpha*, *beta*, *gamma*). The rotation sequence for the Euler angles’ computation was *xz*’*y*’’.

For test A, the relative orientation of each IMMU with respect to itself in the ten repetitions was computed. The standard deviation of Euler angles was computed.

For each repetition of test B and for each distance from the poolside, the relative orientation of each IMMU with respect to IMMU 1, the farthest from the bottom of the pool and then the least affected by the magnetic field inhomogeneity, was computed in order to quantify the orientation error in a slice of the pool.

In tests C and D, the relative orientations of each IMMU between its twelve distances from the metal bar and its largest distance from the metal bar (150 cm) were computed for each repetition. To compare the results of tests C and D, and the differences between the two environments (DL and UW), ANOVA analysis was performed (*p* < 0.05).

#### 2.1.4. Dynamic Orientation Accuracy

**Figure 2:**
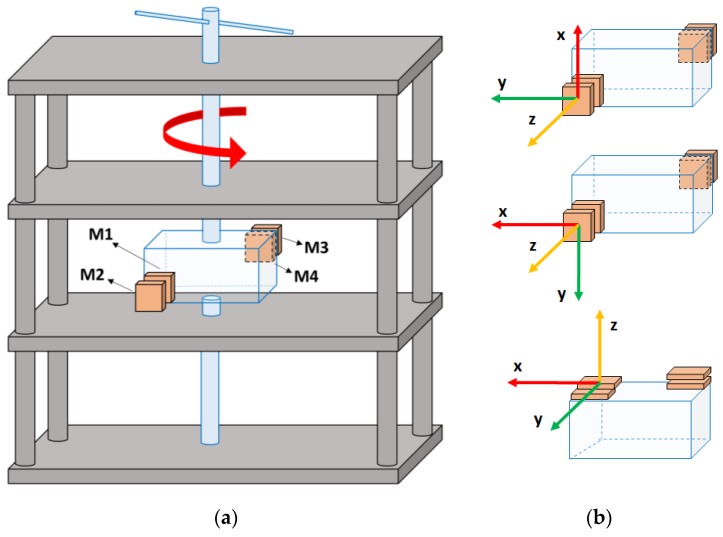
Experimental setup. (**a**) The structure (in gray) is rigidly connected and fixed and has the following dimensions 60 cm× 30 cm × 138 cm. The box is rigidly connected to the central bar (in blue) but free to rotate around the vertical axis. The IMMUs are shown in one of the tested configurations. The whole structure is in a plastic material; (**b**) IMMUs positioning to test all three axis rotations.

The accuracy of the orientation estimation of the IMMUs in the DL and UW settings was compared to validate the use of the IMMUs in the UW setting. To evaluate the dynamic performances of the OPAL system, 4 IMMUs were positioned in pairs, one above the other, and arranged in two vertices of a plastic box. The test, inspired by the works of Cutti et al. [36] and Lebel et al. [40] consisted of a rotation around a single axis at different angular velocities describing 90° arcs in the clockwise and counterclockwise directions alternately. The setup shown in Figure 2a was used to perform the rotation of the box around a single axis. The UW tests were performed in the swimming pool at 1.2 m depth. To stabilize the structure in the water, 8 plastic boxes full of sand (each weighing 2 kg) were positioned on the structure, out of the water. The three different rotations around a single axis were executed by manually spinning the bar rigidly connected to the box around the *x* (RotX), *y* (RotY), and *z* (RotZ) axes of the IMMUs opportunely positioned on the box, Figure 2b. Each rotation was performed with a mean angular velocity of 90°/s (one 90° arc description in one second) and 180°/s (two 90° arc descriptions in the clockwise and counterclockwise directions alternately in one second) and each velocity was maintained using a metronome.

For each speed and each rotation axis, two tests were performed for both DL and UW settings. Each test included 90 s of recording where the first 30 s of the system in static condition were used for the computation of the gyroscope offsets and to allow the Madgwick algorithm to converge, and the remaining 60 s were used to evaluate the dynamic accuracy of the orientation. The value of β used in the Madgwick algorithm for the orientation estimation was 0.312, the value optimized for the gait analysis [32]. The relative orientation of each sensor with respect to each other was computed and expressed in terms of Euler angles (*alpha*, *beta*, *gamma*). For RotX, the rotation sequence for the Euler angle computation was *xz*’*y*’’, for RotY was *yx*’*z*’’, and for RotZ was *zx*’*y*’’. In this way, the first angle *alpha* always corresponded to the direction around which the rotation was performed. Since we performed the rotations around all three axes, we will consider only the alpha angle in further analysis.

Due to the rigidity constraint imposed by the box on the IMMUs, an error-free system would measure a constant angular displacement for each sensor with respect to the others, independently from the motion applied to the box. The IMMUs’ dynamic accuracy can be therefore assessed by measuring the differences of the estimated relative kinematics from this ideal behavior. For all tasks and sensor couples, the range of the displacement of the alpha Euler angle component was measured and it was considered as the measure of the orientation error. Considering all the paired combinations of the 4 IMMUs, 6 couples were obtained. Since we were interested in the overall behavior of the 4 IMMUs, the information relative to the alpha angle was grouped. Each condition (a given rotation, given angular velocity, and a given setting) was thus represented by 36 samples of the error measure of the alpha Euler angle. The dependence of the orientation error on angular velocity (90°/s, 180°/s), rotation direction (RotX, RotY, RotZ), and setting (DL, UW) was evaluated by a 3-factor ANOVA. Multiple comparison post hoc tests were also conducted when the ANOVA found a significant effect. All the statistical analyses were performed with MATLAB and its Statistics and Machine Learning Toolbox (R2016a). A *p* < 0.05 was considered to be significant.

### 2.2. Clinical and Sports Applications

#### 2.2.1. Clinical Application: Gait Analysis

Among the rehabilitation programs, aquatic exercises are frequently recommended for elderly and for patients with compromised, damaged, or impaired posture or locomotion [45], with the aim of improving or at least maintaining gait functions [46,47,48]. Thanks to water’s specific physical properties (density, hydrostatic pressure, buoyancy, viscosity, thermodynamics), participants who perform hydrotherapy feel a reduced weight bearing, augmented drag, and a rise of central venous pressure [45]. Walking is one of the fundamental motor tasks executed during aquatic therapy. Thus, understanding the biomechanics of walking in healthy young adults/elderly is the base for the definition of normative bands and can be compared with pathological patterns of motion. From that starting point, a rehabilitative program in a water environment can be developed. In this section the following main points will be discussed:

(i) The investigation of healthy individuals’ kinematic variables during walking UW using IMMUs, and the definition of normative bands for healthy individuals
(ii) The investigation and comparison of the healthy elderly kinematic variables with those found in younger adults
(iii) The investigation and comparison of the kinematic variables of an anterior cruciate ligament (ACL) injured patient with those found in younger adults.

##### Lower Body Biomechanical Model

The Outwalk protocol [23] was chosen for estimating thorax-pelvis and lower limb kinematics using IMMUs as it entails fast set-up and comfortable calibration procedures with an accuracy comparable to that of optoelectronic systems [49]. Specifically, in the Outwalk protocol, the body is modeled as an open kinematic chain consisting of 8 rigid segments (thorax, pelvis, both thighs, both shanks, both feet) with 21 degrees of freedom. Posterior-anterior tilting, right drop-rise, and right internal-external rotation were calculated for the thorax-pelvis joint. Flexion-extension, abduction-adduction, and internal-external rotation were calculated for the hip and knee joints. Dorsi-plantar flexion, ankle inversion-eversion, and internal-external rotation were calculated for the ankle joint. The thorax-pelvis, the hip, and the ankle were considered as ball-and-socket joints, while the knee was considered as a ‘loose’ double-hinge joint.

The anatomical reference systems (ARSs) were computed through two types of trials: (A) a static calibration with the participant standing in an upright posture; (B) a functional calibration with the participant performing five knee flexion-extensions up to 70° of flexion at a self-selected speed. While the first calibration established mainly the joint angle offsets of the hip and the ankle, the second calibration allowed for the orientation estimation of the mean flexion-extension axis of the knee. More details regarding the protocol can be found in Cutti et al. [23].

The following indications on positioning the IMMUs on the participant’s body were followed to ensure the maximum accuracy of the protocol and the reduction of the soft-tissue artifacts. The IMMU on the thorax was placed in the middle area between the incisura jugularis and processus xiphoideus, aligning the x-IMMU axis to the long axis of the sternum. The IMMU on the pelvis was placed with the x-IMMU axis aligned with the left-right axes line. The IMMUs on the thighs were placed in the central-third, with the z-IMMU axis pointing laterally. The IMMUs on the shanks were placed slightly above the lateral malleolus, with the z-IMMU axis pointing perpendicular to the sagittal plane. The IMMUs on the feet were placed over the flat portion of the lateral part of the metatarsal area.

##### Set-Up

Eight IMMUs (Opal, APDM) were used. The acquisition frequency of each IMMU was set to 128 Hz and the accelerometer’s full scale was set to ±6 g. The IMMUs were calibrated at the beginning of each acquisition session, inserted in round plastic waterproofed boxes and fixed to the body segments of the participant by means of elastic bands. Furthermore, adhesive tape and adhesive spray were used to ensure that the boxes remained fastened to the skin.

##### Participants

Healthy young adult participants. Six males and five females, (27.0 ± 3.4 years, 174.2 ± 8.2 cm height, 70.2 ± 11.8 kg mass) free from known musculoskeletal, neurological, cardiac, or pulmonary diagnosis participated in the study

Healthy elderly participants. Three males and two females (71.6 ± 2.2 years, 167.8 ± 6.9 cm height, 67.0 ± 13.0 kg mass) free from known musculoskeletal, neurological, cardiac, or pulmonary diagnosis participated in the study.

Pathological participant. A male (39 years old, 171 cm height, 85 kg mass) with a complete tear of his left ACL was acquired. Before the surgery, the ACL knee could reach a maximum flexion of about 20°, though the extension was not compromised. Twenty-eight days after the injury the patient had a successful reconstructive surgery of the ACL left knee. A graft was harvested from the patient hamstring tendons and was inserted into femoral bone tunnels to replicate his native ACL. The session test was then performed 45 days after the surgery.

All the participants gave written informed consent to participate in the acquisitions, approved by the Bioethics Committee of the University of Bologna for both the young adults group and for the elderly groups.

##### Motor Task

All the participants were measured during walking in water (1.2 m depth at a temperature of 28 °C) and during walking on land setting for three 10 m walking barefoot trials at a self-selected comfortable speed.

##### Data Analysis

The IMMUs estimation of the orientation was computed by combining raw data from the gyroscopes, accelerometers, and magnetometers through the Madgwick filter. The β gaining factor defined by Madgwick was previously tuned for the specific motor task, comparing inertial data with optoelectronic data in laboratory controlled conditions (BTS Smart-DX 7000). The optimal β was 0.312.

Gait cycles were automatically segmented [50] and selected if the relevant Intraclass Correlation Coefficient value was higher than 0.75 [32]. Firstly, spatiotemporal parameters, walking speeds (cm/s), stride times (s), and stride lengths (cm) were computed [50]. The data analysis was then completed computing angular parameters for all the participants, their median, as well as the 25th and 75th percentiles. The joint angular kinematics of the lower limbs were calculated at heel-strike and at toe-off. Finally, for each joint angle, the maximum, minimum, and Range of Motion (ROM) were calculated. One way non-parametric ANOVA test was performed to evaluate significant differences between young adult, elderly, and pathological participants. All the statistical analyses were performed with the MATLAB Statistics and Machine Learning Toolbox (R2016a).

#### 2.2.2. Sport Applications: Swimming Analysis

Quantitative and objective technical evaluation of swimming is an essential factor for swimmers’ training. The use of IMMUs may allow for improved analysis of stroke mechanics, of performance and of energy expenditure, as well as real-time parameters/variables available for the coaches, who can potentially provide the swimmers with more efficient, precise, and objective feedback. Different types of analyses can be performed depending on the coaches/athletes questions to be answered. Hereinafter, the two main investigations that can be performed using IMMUs in swimming will be described:

(i) Temporal phases detection
(ii) Upper limb joint kinematic analysis

##### Upper Body Biomechanical Model

The protocol proposed by Cutti et al. [51] and slightly adapted to the sport context by Fantozzi et al. [33] was used for estimating upper limb kinematics using IMMUs. Specifically, each upper limb is modeled as an open kinematic chain consisting of 4 rigid segments (thorax, upper-arm, forearm, and hand) with 7 degrees of freedom. Similarly to the model of Cutti et al. [51], the shoulder was considered as a ball-and-socket joint, while the elbow and the wrist were considered as double-hinge joints.

The ARSs were computed through three types of trial: (A) a static calibration with the participant lying in the supine position, keeping the arms alongside the body and holding the dorsum of the hands aligned to the upper side of the forearms; (B) a functional calibration with the participant performing 10 elbow flexion-extensions, from about 10° to 100° of flexion, keeping constant pronation-supination; (C) a functional calibration with the participant performing 10 full-range elbow pronation-supinations keeping constant flexion-extension. While the first calibration established mainly the joint angle offsets of the shoulder and the wrist, the second and third calibrations allowed for the orientation estimation of the mean flexion-extension and prono-supination axes of the elbow. Further details of the protocol can be found in Fantozzi et al. [33].

To perform the alignment between the IMMU reference systems and the ARS defined by means of the calibration procedure described above, a specific positioning of the IMMUs on the body segments is required. The unit on the thorax was fixed by aligning the IMMU *X*-axis to the longitudinal axis of the flat portion of the sternum. The unit on the humerus was fixed laterally, slightly above the center of the humerus and posteriorly. The unit on the forearm was fixed distally, just above the ulnar and radial styloids, with the IMMU *Z*-axis pointing away from the wrist. The unit on the hand was fixed over its dorsum, with the IMMU *Z*-axis pointing away from the hand.

##### Set-Up

Seven IMMUs (Opal, APDM) were used. The acquisition frequency of each IMMU was set to 128 Hz and the accelerometer’s full scale was set to ±6 g. The IMMUs were calibrated at the beginning of each acquisition session, inserted in round plastic waterproofed boxes, and fixed to the body segments of the participant by means of elastic bands. Adhesive, tape, and adhesive spray were used to ensure that the boxes remained fastened to the skin.

##### Participants and Motor Task

Six male regional-level swimmers (26.1 ± 3.4 years; 182.5 ± 8.8 cm height; 77.0 ± 10.1 kg mass) were recruited to take part in the study. All the participants gave written informed consent to participate in this study that was approved by the Bioethics Committee of the University of Bologna.

Initially, they were asked to perform simulated front-crawl swimming at self-selected speed and stroke. Each participant performed simulated swimming, in the DL setting, after leaning the lower limbs (held by an operator) against a 70 cm high plastic rigid box, and having the pelvis, trunk, and upper limbs free to move. Twenty complete strokes were acquired, from both IMMUs and stereo-photogrammetry, for each swimmer, after a familiarization trial. Successively, the participant performed front crawl in the UW setting at self-selected speed in a 25 m swimming pool. For each participant, three trials of 50 m were acquired.

##### Data Analysis

Temporal phases detection. The segmentation of the simulated swimming stroke cycles was then performed to develop a semi-automatic algorithm that recognized the maxima of the elbow flexion-extension angle and the minima of the shoulder flexion-extension angle of the front-crawl [33]. The detection of the front-crawl temporal phases was performed using the algorithm proposed by Dadashi et al. [52] which is based on a Kalman adaptive filtering of inertial signals recorded from sensors positioned on both the forearms and on the sacrum. According to video analysis of Chollet et al. [53], four arm phases per stroke (entry and catch, pull, push, recovery) were determined. Entry and catch correspond to the times when the hand enters into the water until the beginning of its backward movement; this phase includes more or less the time spent to glide with the arm extended forward. The pull phase begins at the forearm backward movement ($T_{PUL}$) until its entry to the transversal plane crossing the shoulders. The push phase is the time between the positioning of the hand below the shoulder ($T_{PUS}$) and its exit from the water. The recovery phase corresponds to the time interval between when the hand exits the water ($T_{REC}$) and its forthcoming entry to the water. The algorithm proposed by Dadashi et al. [52] allows the identification of three of those phases: pull, push, and recovery phases. Starting from these phases, the pull duration $∆t_{PUL}$, the push duration $∆t_{PUs}\;$, and the non-propulsive interval $∆t_{NPROP}$ were extracted as in Equations (1)–(3) [52]:

(1) $$∆t_{PUL}^{k}=t_{PUS}^{k}-t_{PUL}^{k}$$

(2) $$∆t_{PUs}^{k}=t_{REC}^{k}-t_{PUS}^{k}$$

(3) $$∆t_{NPROP}^{k}=t_{PUL}^{k}-t_{REC}^{k-1}$$

Stroke rate SR was also extracted from the acquired signals. The temporal phases were detected only for the data acquired during the front crawl trials in the UW setting, thus the detection was applied only for the setting where the algorithm was previously validated [52].

Joint kinematic analysis. The IMMUs estimation of the orientation was computed by combining raw data from the gyroscopes, accelerometers, and magnetometers through the Madgwick filter. Two different β values, for all the calibrations (0.6250) and the front-crawl (0.8750) trials, respectively, were obtained by minimizing the root-mean-square error (RMSE) between the IMMUs and stereo-photogrammetric system, averaged among all the trials for all the units/clusters, for all the participants.

For the DL simulated swimming, the kinematic measurements obtained with the IMMUs were compared to those of the stereo-photogrammetric system, using coefficient of multiple correlation (CMC) [54], Pearson product-moment correlation coefficient (R), RMSE, and percentage ratio between RMSE and the relevant range of motion (rRMSE). The analyses were performed using the statistical software R (version 3.0.2) Further details can be found in [33].

## 3. Results

### 3.1. Instrument Validation

#### 3.1.1. Magnetic Field Mapping

The results of test A showed that the measures were repeatable (maximum standard deviation = 0.9°), thus the results obtained in the following tests can be generalized.

Recordings from the IMMUs showed that the IMMU orientation estimation was strongly dependent on the distance between the metal bar and the IMMUs, both in the lab and in the swimming pool, which clearly indicated that the magnetic field was not homogeneous. The comparison between tests C and D revealed a significant difference (*p* < 0.05). Specifically, the mean orientation error in DL was lower than that in the UW settings. The orientation errors estimated in DL, moving a ferrous object toward the IMMUs, decayed exponentially and were smoother than those in UW, moving the same object toward the IMMUs. The orientation errors in UW setting, although with exponential decays, were noisier and less stable and remained greater than those in DL.

**Figure 3:**
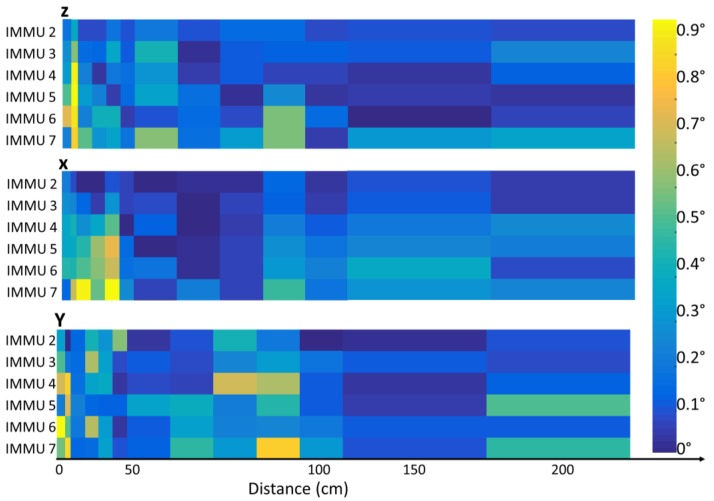
Relative static orientation error (deg) due to the swimming pool side and bottom measured by the IMMU at 250 cm from the beginning of the lane and at different distances (test B) from the pool side with respect to IMMU 1, the furthest IMMU from the bottom of the pool.

**Figure 4:**
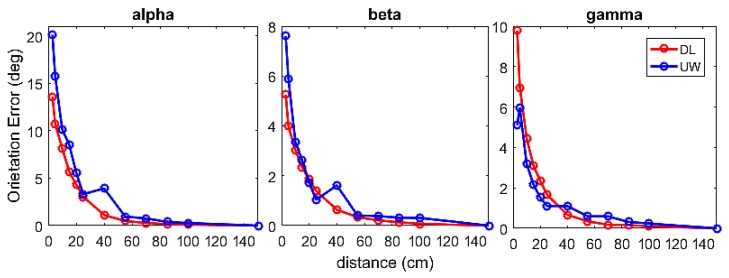
Relative static orientation errors (deg) averaged on all the IMMUs (IMMU1–IMMU6) and on the three repetitions of the tests C and D. The orientation error is showed as function of the difference from the metal objects in the DL (red lines, test C) and UW (blue lines, test D) settings.

The results of test B, (displayed in Figure 3), and those of tests C and D (Figure 4), showed that the strongest effects appeared at 3 cm near the bottom of the swimming pool side (relative orientation error up to 0.9°) or near a metal object (relative orientation error up to 20°, Figure 4). At 100 cm from the swimming pool side, the relative orientation error was lower than 0.6° for all the IMMUs. At 100 cm from a metal object, the relative orientation error became lower than 0.5° for both DL and UW settings.

#### 3.1.2. Dynamic Orientation Accuracy

**Figure 5:**
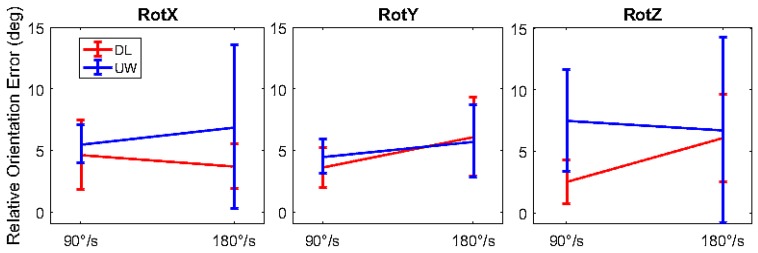
Orientation error (range of the Euler angles) for the three rotational directions, for the two different velocities and for the two settings (DL and UW). Mean values and SD are represented.

The results shown in Figure 5 revealed that the error of measurement depended on the factor’s angular velocity (*p* < 0.05) and settings (*p* < 0.05). Specifically: (i) the error generally increased with the angular velocity and (ii) the UW setting provided a larger error than that of the DL setting. For the DL setting a mean error of 4.4° was found, while for the UW setting a mean value of 6.1° was found. Thus, the difference in performance between the DL and UW settings was about 2°.

### 3.2. Clinical and Sport Applications

#### 3.2.1. Clinical Application: Gait Analysis

##### Healthy Young Adult Participants

**Figure 6:**
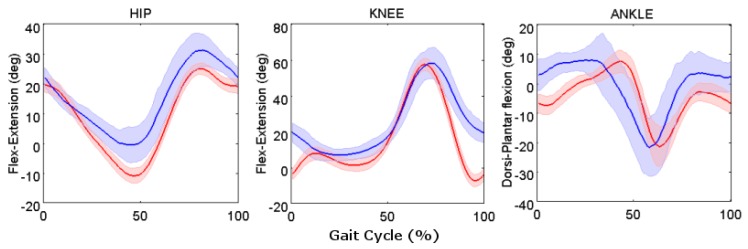
Angular kinematic patterns of the lower limb joints (hip, knee, and ankle) in the sagittal plane. Median, 25th, and 75th percentiles for all the participants for the DL (red solid line and shaded area) and UW (blue solid line and shaded area) settings. The gait cycles are normalized in time.

The first analysis on spatiotemporal parameters and walking speed showed a reduction of 40% of the median speed in water with respect to that of the DL setting (*p* < 0.05). Furthermore, an increase of the median stride duration of 60% and a decrease of 7% of the median stride distance were found in the UW walking (*p* < 0.05) setting [32]. Regarding the kinematic variables, in UW settings, the knee was more flexed, the ankle was more dorsiflexed (≈9°) at heel strike, and the hip was more flexed at toe-off (≈13°) than on land walking (Figure 6). Further details can be found in [32].

##### Healthy Elderly Participants

In elderly participants, the median stance duration expressed in percentage of stride cycle was 5% higher than in young adults (*p* < 0.05). Whereas, both the median swing duration and duration percentage were slightly decreased (14% and 7% respectively; *p* < 0.05). On the contrary, both the median stride length and the median walking speed were strongly decreased (49% and 54% respectively; *p* < 0.05) in elderly participants than in young adults.

**Figure 7:**
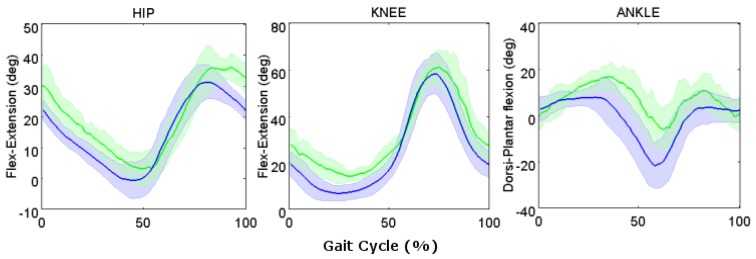
Angular kinematic patterns of the lower limb joints (hip, knee, and ankle) in the sagittal plane. Median values, 25th, and 75th percentiles for all the participants for the young healthy participants (blue solid line and shaded area) and elderly healthy participants (green solid line and shaded area) in the UW settings. The gait cycles are normalized in time.

Looking to the kinematic variables, similar patterns but with some peculiar differences were found between young adults and elderly participants in the UW setting in the sagittal plane (Figure 7). More specifically, looking at the sagittal plane at heel strike, the knee was found to be more flexed (by about 8° comparing the mean values) and the ankle more dorsiflexed (by about 8°) in the elderly (Figure 2) compared to young adult participants (*p* < 0.05). Regarding the stance, the knee showed higher values of flexion in elderly participants than young adults, with a difference between the minimum values of 7° (*p* < 0.05). The ankle showed higher values of plantar flexion from the mid stance through most of the swing phase for the elderly participants with a difference of about 15° at toe-off (*p* < 0.05). In the swing phase, the hip and the knee were more flexed also during the terminal-swing phase of about 8° and 10°, respectively (*p* < 0.05).

##### Pathological Participant

**Figure 8:**
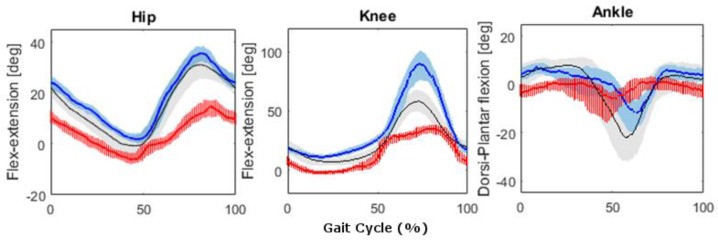
Angular kinematic patterns of the lower limb joints in the sagittal plane. Median, 25th, and 75th percentiles in UW setting, of the injured (red solid line and red stripes area) and the contralateral (blue solid line and light blue shaded area) limbs of the participants. The healthy young adult patterns are indicated with the black line and gray shaded area. The gait cycles are normalized in time.

In the following, the results obtained in the UW setting will be shown. Complete details can be found in [55]. The gait patterns of the patient were different with respect to those of the healthy young adults and comparing the injured side with the contralateral one (Figure 8). The patient showed both a reduced maximum flexion and a reduced Range of Motion (ROM) (36° with respect to 56° and 39° with respect to 60°, respectively) of the injured knee with respect to those of the young adults. The overall knee ROM of the injured knee was 47% of that of the contralateral knee, and the overall hip ROM was 64% of that of the contralateral hip (*p* < 0.05). Finally, a reduced ROM was also observed for the ankles with a smaller value at the injured limb with respect to that of the contralateral one.

#### 3.2.2. Sport Applications: Swimming Analysis

##### Temporal Phases Detection

**Figure 9:**
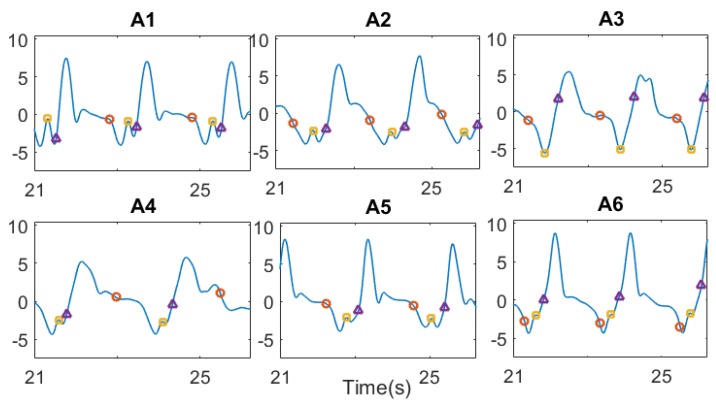
Examples of the gyroscope data output recorded from the right forearm for the six athletes (A1–A6). The three time events, $T_{PUL}$ as the red circle, $T_{PUS}$ as the yellow square, and $T_{REC}$ as the purple triangle, are shown in the figure.

Figure 9 shows the patterns of the right forearm gyroscope data with superimposed time events detected with the Dadashi algorithm [52]. Differences in the gyroscope pattern were observed among the six athletes. A1 and A5 showed a distinct plateau in the part of the curve between the maximum and the minimum allowing a clear and repeatable detection of $T_{PUL}$ (red circle in Figure 9). The remaining athletes in one case exhibited no plateau at all (A2) and in other cases had a short and variable duration of the plateau (A3, A4, and A6) resulting in an irregular detection of $T_{PUL}$. Similar observations can be drawn for $T_{PUS}$ (yellow square in Figure 9) as not all the athletes analyzed showed a clear and detectable small peak after the minimum; for example, A3 exhibited no peak at all.

The performance indices extracted for the front-crawl swimming are shown in Table 2. Considering the mean values of $∆t_{PUL}$, the athletes exhibited comparable results, except for A4. This difference is linked with the different pattern of the gyroscope data output of S4, determining an anticipation of the detection of $T_{PUL}$ from the algorithm and, as a consequence, an extra time of $∆t_{PUL}$. Four athletes (A2, A3, A4, and A6) out of six showed larger standard deviations of $∆t_{PUL}$. No differences among the athletes were highlighted in the mean values of $∆t_{PUS}$ and $∆t_{NPROP}$. Two athletes (A4 and A6) out of six showed larger standard deviations of $∆t_{PUS}$. The variability among athletes for SR were in line with the unconstrained instructions given during the execution of the 50 m front-crawl.

##### Joint Kinematic Analysis

In simulated front-crawl swimming, when comparing IMMUs results with stereo-photogrammetry results, the overall median (first–third quartiles in brackets) values were 0.97 (0.93–0.99) for CMC, 0.95 (0.93–0.98) for R, 7° (4°–10°) for RMSE, and 9% (6%–11%) for rRMSE. Regarding the different joints, the angles estimated using the IMMU system showed a better agreement with those estimated using the stereo-photogrammetric system in proximal joints. Further details can be found in [33].

**Figure 10:**
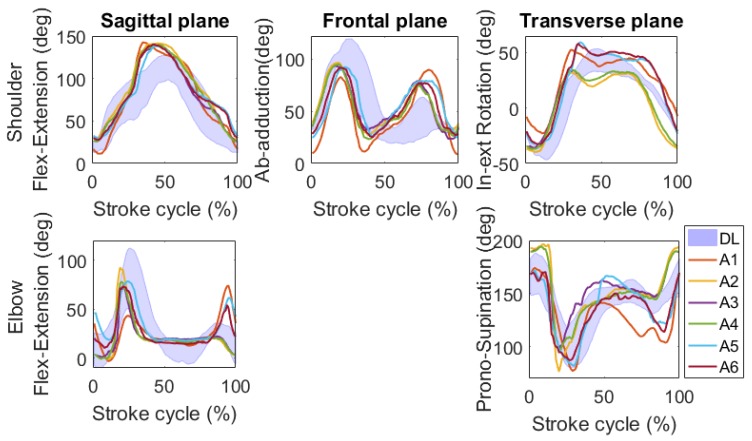
Shoulder and Elbow joint angle median values on all the stroke cycles, computed using IMMUs relative to the sagittal, frontal, and transversal plane of front-crawl swimming for the six participants. The stroke cycles are normalized in time. Simulated front-crawl bands are shown for comparison.

Based on the results obtained in simulated swimming, an analysis of the joint kinematics of front-crawl in a 25 m swimming pool was then carried out. Figure 10 shows the shoulder and elbow joint angle median values relative to the sagittal, frontal, and transversal plane of the front-crawl swimming, obtained using the IMMUs for the six participants and the validated pattern of the same athletes in simulated swimming. Regarding the shoulder joint, no differences among athletes was observed on the sagittal and frontal planes. Whereas on the transverse plane from 40% to 100% of the stroke cycle, A2 and A4 exhibited a shoulder more internally rotated with respect to the other participants (A1, A3, A5, and A6). Regarding the elbow joint, similar patterns and values among participants were observed during the propulsive phase (from about 40% to about 80% of the stroke cycle) on the sagittal plane. Whereas during the non-propulsive phase on the sagittal plane and during the entire cycle on the transverse plane, athletes exhibited different patterns of motion.

Comparing the motor patterns between the DL and UW settings, the main differences were found in the sagittal and frontal planes. In the sagittal plane, the shoulder showed the maximum flex-extension value in UW greater than that in the DL settings (≈26°). On the contrary, the elbow showed the maximum flex-extension value in UW lower than that in the DL settings (≈10°) in the air phase (the phase that begins at 0% of the stroke cycle and ends at the maximum shoulder flex-extension in Figure 10), and a greater flexion in the underwater phase (the phase from the maximum shoulder flex-extension to 100% of the stroke cycle) of the stroke cycle. In the frontal plane, the shoulder ab-adduction showed the maximum flex-extension value in UW lower than that in the DL settings (≈10°) in the air phase and a much greater flexion in the underwater phase (34°) of the stroke cycle in UW than that in the DL setting.

## 4. Discussion

The aim of the present study was two-fold: the instrumental validation of IMMUs in water, and the description of their use in clinical and sports aquatic applications using multi-body kinematics analysis.

### 4.1. Instrument Validation

The use of IMMUs was validated in water and some peculiarities were highlighted to allow a reliable collection of data for the experimental applications.

#### 4.1.1. Magnetic Field Mapping

Several tests were performed to quantify the effects on the orientation estimation of the magnetic field distortion introduced by ferrous objects in the laboratory and by the swimming pool wall and floor. In the tested conditions, the static orientation accuracies are in line with the results of Cutti et al. [36] for a test performed in a typical movement analysis laboratory at a distance of at least 200 cm from ferromagnetic objects. A significant difference was found between the DL and UW environments, with a higher static orientation error in the last setting. The comparison between DL and UW settings discloses a different magnetic field distortion, induced by the same ferrous object, between the water and the air, however producing an orientation estimation error lower than 0.5° 100 cm from the metal object.

These tests allowed us to define a set of guidelines about the position in which to perform the experimental acquisitions in order to have a homogeneous magnetic field that does not affect the orientation estimation in water: (i) perform the experiments in the laboratory and in the pool at a distance of at least 100 cm from ferrous objects and (ii) perform the experiments in the pool at a distance of at least 100 cm from the poolside.

#### 4.1.2. Dynamic Orientation Accuracy

The dynamic orientation error has been shown to depend on both the angular velocity and the setting. We found that the error increases with the increase of the angular velocity, in accordance with Cutti et al. [36] and Label et al. [40]. The dynamic orientation error in water was quantified and compared with that in the DL condition. The obtained results are in line with the results of Cutti et al. [36] for the DL setting with a mean error of 4.4°. The UW setting provided a greater error than that in DL, which can be explained by the different distribution of the magnetic field distortion in the water, as also found in the static orientation error. However, since the UW mean dynamic orientation error was 6.1°, the difference in performance between the DL and UW settings was lower than 2°. For these reasons, in the experimental application analysis, only variations larger than 6° should be considered.

As shown by Lebel et al. [40], the analyzed conditions, i.e., rotation around a single axis and relative orientation accuracy estimation, provide worse performance than mixed rotations and absolute accuracy estimation. Since gait and swimming tasks are characterized by mixed rotations and by lower angular velocity (than those of the tested conditions), we retain that orientation errors are lower as well.

### 4.2. Clinical and Sport Applications

#### 4.2.1. Clinical Application: Gait Analysis

After the instrumental validation, the main clinical application using multi-body joint kinematics in aquatic therapy was shown. The gait analysis in water was explored for young adult, elderly, and pathological participants, in terms of spatiotemporal and lower limb joint kinematics parameters.

##### Healthy Young Adult Participants

The spatiotemporal analysis showed the following main results: (i) reduction of median speed; (ii) longer stride duration; and (iii) shorter stride distance in water with respect to DL. These results confirmed the results presented in the literature using video cameras [39,56,57].

Regarding the kinematic variables, differences between DL and UW were found in flexion-extension of both the knee and ankle at heal strike and of the hip at toe-off. These findings are controversial in studies that used video cameras: some studies [58] found results similar to those we presented, while others [59] observed no differences at heel-strike and toe-off for healthy young adults. The differences found in the present paper can be explained by the surrounding conditions, i.e., walking speed of the participants and/or their height. This hypothesis is supported also by the results of the linear mixed model analysis performed [32,60]. Thus, it seems that the combination of speed and environment (on land or UW) triggered modifications in the joint angles in UW gait more than these two factors considered separately.

Lastly, the analysis of young adults allowed the definition of normative bands.

##### Healthy Elderly Participants

The preliminary results obtained for the elderly participants showed an increased median stance duration percentage with respect to that of young adults and a decreased median swing duration and duration percentage in accordance with the results of Barela et al. [39] obtained with video recording. On the contrary, the median stride length and the median walking speed were strongly decreased in contrast with the results of Barela et al. [39]. These results can be explained by the different performance of both the elderly and young adults on DL with respect to the study of Barela. Specifically, young adult participants of the present study showed higher walking speed and stride length while elderly participants showed lower walking speed and stride length.

The difference found in knee and hip flexion-extension between the elderly and young adults could be explained by the effect of the walking speed in the elderly participants. For this reason, this aspect will be a matter of future investigation on a larger group of elderly participants. The kinematic analysis revealed an increase of the knee and hip flexion and a decrease of the plantar flexion in elderly participants with respect to young adults. These differences were shown by Kerrigan et al. [61], using stereophotogrammetry for walking on land, and were associated to both subtle hip flexion contracture and ankle plantar flexor concentric weakness of the elderly participants. The present preliminary results, in agreement with Barela et al. [39], showed specific UW gait patterns for elderly participants, highlighting the importance of using different joint angle normative bands accordingly to the age.

Further data will be acquired to define normative bands on a larger sample of participants.

##### Pathological Participants

The spatiotemporal analysis showed no differences between the injured and the contralateral sides. However, different joint kinematic variables were found. This result confirms the need of using 3D joint kinematics variables to have a deeper understanding of the patient biomechanics. Furthermore, the analysis of the pathological participant enhanced the feasibility of the method in providing quantitative gait data, in both on land and UW settings, that could be used to monitor the treatment evolution and to objectively support decisions of the clinicians.

#### 4.2.2. Sport Application: Swimming Analysis

Finally, the application of the IMMUs in aquatic sports was described. The swimming analysis was explored in term of spatiotemporal and upper limb joint kinematics variables.

##### Temporal Phases Detection

The obtained results are in line with the performance indices extracted using video camera recordings by McCabe et al. [9] and by Cortesi et al. [8], on both phase duration and stroke rate. However, some critical issues should be highlighted. The application of the Dadashi algorithm [52] in UW settings showed contrasting results among the participants because of their different stroke patterns in terms of angular velocity, as shown in Figure 9. Specifically, $T_{PUL}$ events were the most critical to be identified as shown by their high standard deviation reported in Table 1. Another limitation of that algorithm is that it does not allow the detection of the entry and catch events, limiting the performance indices that can be extracted (i.e., index of coordination [62]). The detection of the stroke phases through the hand trajectory estimation could represent a more robust method to enable the recognition of different swimming patterns, and could allow for the detection of the entry and catch events, thus overcoming the described limitations. Furthermore, the spatiotemporal parameters are considered to be not exhaustive at describing the motor gesture.

##### Joint Kinematics Analysis

The joint kinematic information, relevant to the shoulder and elbow, has shown to be an added value of great interest in the analysis of swimming, for coaches and athletes. The findings of the simulated swimming represent an important step towards the practical use of technology based on IMMUs for the kinematic analysis of swimming in applied contexts.

Based on the results obtained in simulated swimming, an analysis of the joint kinematics of front-crawl in a 25 m swimming pool was then carried out. Even if no validation was carried out in the UW joint kinematic analysis, the results of the instrumental validation proposed in Section 2, allow for the application of the model and the type of analysis validated for DL settings also in UW settings, during front crawl. The main difference between the simulated and UW front-crawl was a greater ab-adduction of the shoulder in the frontal plane during the underwater phase of the stroke cycle. The pattern of the UW setting is the typical pattern of a curvilinear arm pull, whereas the pattern of the DL setting is typical of a straight arm pull. The curvilinear arm trajectory is due to the drag and lift forces of the water that the athletes have to create for the generation of a more efficient hand propulsion, so it is not visible in DL [63].

The results obtained in front-crawl demonstrate the feasibility of the joint kinematic analysis in water. The appropriateness of the experimental setup and of the biomechanical model used is supported by the results found in the literature for video camera recordings [64] that are in line with the present results.

The next steps in the analysis of swimming using IMMUs will be the comparison between different swimming conditions (e.g., free or tethered), different level of swimmers, and the correlation of the 3D-joint kinematics with the performance indices typically used by the coaches during training sessions.

The results obtained in the DL simulated swimming and in the front-crawl UW swimming represent an important step forward for demonstrating the reliability and the feasibility of the joint kinematic estimation using IMMUs.

## 5. Conclusions

The reported applications demonstrated the potentiality of the IMMUs in the water environment.

The instrumental tests quantified a dynamic orientation estimation accuracy of about 6°. This accuracy is lower than that obtained with the standard stereophotogrammetric system, however it is considered enough to provide useful information about the two main fields of interest described in this paper, gait and swimming. The obtained results highlight the potentiality of the IMMU in the comparison between young healthy, elderly, and pathological participants during gait and in the comparison between different swimming styles.

The use of the IMMUs also provided several advantages over more expensive and bulky systems, such as (i) simpler and faster setup preparation; (ii) less time consuming processing phase, and (iii) the chance to record and analyze a higher number of strides/strokes without limitations imposed by the camera’s volume of acquisition.

## Figures and Tables

**Table 1:** Technical specification of the used IMMU (Opal, APD, USA).

	Accelerometer	Gyroscope	Magnetometer
Range	±2 g; ±6 g	±2000°/s	±6 Gauss
Bandwidth	50 Hz	50 Hz	50 Hz
Resolution	14 bit	14 bit	14 bit
Noise	128 μg/$\sqrt{Hz}$	0.07°/s/$\sqrt{Hz}$	4 m Gauss/$\sqrt{Hz}$

**Table 2:** Performance indices of the six athletes (A1–A6) extracted from the front-crawl swimming trials. Results are showed as mean ± std computed over the three trials for about 50 stroke cycles.

	$∆t_{PUL}\;\left(s\right)$	$∆t_{PUS}\;\left(s\right)$	$∆t_{NPROP}\;\left(s\right)$	$SR\;\left(cycle\;m^{-1}\right)$
A1	0.43 ± 0.06	0.24 ± 0.06	1.34 ± 0.06	43.80 ± 0.60
A2	0.54 ± 0.22	0.30 ± 0.05	1.00 ± 0.21	45.00 ± 1.62
A3	0.54 ± 0.26	0.34 ± 0.05	1.14 ± 0.26	55.80 ± 0.60
A4	0.79 ± 0.24	0.21 ± 0.12	1.53 ± 0.21	32.42 ± 1.22
A5	0.42 ± 0.09	0.25 ± 0.09	1.49 ± 0.12	41.43 ± 0.54
A6	0.30 ± 0.15	0.25 ± 0.12	1.53 ± 0.14	43.27 ± 0.48
